# Highly Active and Stable Fe-N-C Oxygen Reduction Electrocatalysts Derived from Electrospinning and In Situ Pyrolysis

**DOI:** 10.1186/s11671-018-2635-x

**Published:** 2018-07-20

**Authors:** Xuelian Yan, Yucen Yao, Yuan Chen

**Affiliations:** 1 Research Institute for New Materials Technology, Chongqing University of Arts and Sciences, Yongchuan, Chongqing, 402160 People’s Republic of China; 2 Suzhou Institute for Energy and Material Innovations, Soochow University, Suzhou, 215006 China

**Keywords:** Electrocatalysts, Oxygen reduction reaction, Fe-N-C

## Abstract

High-performance electrocatalysts for the oxygen reduction reaction (ORR) are essential in electrochemical energy storage and conversion technologies. Fe-N-C electrocatalysts have been developed as one of the most promising alternatives to precious metal materials. Current M-N-C electrocatalysts usually are derived from high-temperature thermal treatment of a nitrogen-containing polymer or metal–organic frameworks (MOFs). Here, we developed Fe-N-C mesoporous nanofibers with low-cost urea and FeCl_3_ as the nitride and iron source; the electrocatalysts with abundant Fe-Nx active sites and large surface area were synthesized via electrospinning, in situ pyrolysis, and acid treatment process. The use of sealing conditions in the calcination process can effectively improve the nitrogen species content in the catalyst, which is important for improving performance. The as-prepared electrocatalyst material manifests well electrocatalytic performance for ORR in alkaline electrolyte (onset potential of 0.93 V and half-wave potential of 0.82 V); meanwhile, the electrocatalyst expresses good stability and methanol tolerance. This work may provide new thought for developing high-performance ORR electrocatalysts.

**Electronic supplementary material:**

The online version of this article (10.1186/s11671-018-2635-x) contains supplementary material, which is available to authorized users.

## Background

Fuel cells are of tremendous interest for clean energy conversion devices, and the oxygen reduction reaction (ORR) is the major limiting factor [1]. Platinum-based electrocatalysts have been considered as the most effective catalysts for ORR, but they are still seriously restricted by the issues concerning their high cost, insufficient durability, crossover effect, CO poisoning, and limited reserve in nature [2, 3]. Developing nonprecious metal catalysts with high ORR performance to replace Pt-based catalysts for practical applications is necessary. In this regard, a plenty of works, including transition metal and nitrogen co-doped carbons (M–N/C, M = Fe, Co, Ni) [4–8], metal-free heteroatom-doped carbons [9–11], and metal oxide-carbon composites [12, 13], have been reported for replacing Pt-based catalysts. Among these candidates, the Fe-N-C emerged as the most potential one due to their excellent activity and stability for ORR [4–6].

Currently, researchers have been reported that the excellent ORR performance in Fe-N-C catalysts was derived from the nitrogen-coordinated iron sites (Fe-Nx) embedded in the basal planes of carbon [14, 15]. Density functional theory (DFT) calculations show that the configuration of Fe-Nx significantly affects the electronic structures of the Fe center, which further affects the binding energy of reactants (O_2_), products (H_2_O), and intermediates (e.g., H_2_O_2_, OOH*, and OH*) with the Fe center, thereby leading to variations in electrocatalytic activity [16, 17]. To obtain high-performance Fe-N-C ORR catalysis, it should be devoted to construct abundant Fe-Nx sites. The most direct way was pyrolysis complexes containing Fe–N_4_ moieties or metal–organic frameworks (MOFs); however, they were obtained by complex reaction process. In addition, carbon support morphology and pyrolysis temperature also affect the active site exposure and the conductivity which further determine the electrocatalyst performance.

In this work, we developed Fe-N-C mesoporous nanofibers with low-cost urea and FeCl_3_ as the nitride and iron source; the electrocatalysts with abundant Fe-Nx active sites and large surface area were synthesized via electrospinning, in situ pyrolysis, and acid treatment process. The use of sealing conditions in the calcination process can effectively improve the nitrogen species content in the catalyst, which is important for improving performance. The Fe-N-C catalysts exhibit high ORR activity in alkaline media; it also demonstrated remarkable stability and methanol tolerance.

## Methods

### Synthesis of the Fe-N-C Mesoporous Nanofibers

All chemicals in the experiment were used without any further purification. In a typical experiment, 0.8 g polyacrylonitrile (PAN; Mw = 150,000), 0.1 g FeCl_3_, and 0.5 g urea were dissolved in 10 mL *N*-*N*-dimethylformamide (DMF) under vigorous stirring for 6 h to form a homogeneous solution. For a typical electrospinning process, the spinneret diameter was 0.9 mm; a distance of 15 cm and a direct current voltage of 18 kV were maintained between the tip of the spinneret and the collector. After electrospinning, the obtained fibers were collected and then maintained in a tube furnace at 800 °C for 2 h. It should be noted that to avoid the N volatile at high temperature, a lid was added to the top of the porcelain boat. After that, the product was immersed in HCl for 5 days to remove the redundant iron. Finally, the Fe-N-C porous nanofibers were obtained, and it was named FN-800.

### Instruments

The as-prepared sample was characterized by X-ray powder diffraction (XRD; Philips X’Pert Pro Super diffractometer, *λ* = 1.54178 Å), transmission electron microscopy (TEM; Tecnai G20), field emission scanning electron microscopy (FE-SEM; Hitachi, SU 8010), energy dispersion spectra (EDS; JEOL JEM-ARF200F), nitrogen adsorption−desorption isotherms (Micromeritics ASAP 2000); X-ray photoelectron spectra (XPS; ESCALAB MK II), and Raman spectroscopy (HR 800 Raman spectrometer, Jobin Yvon, Horiba, France) using a 532-nm green laser.

### Electrochemical Measurements

All the electrochemical measurements were performed in a three-electrode system on an electrochemical workstation (CHI660B). Firstly, 5 mg of catalysts and 150 μL of 5 wt% Nafion solutions (Sigma-Aldrich) were dispersed in 350 μL of ethanol solution with sonication for 30 min to form a homogeneous ink. The 5 μL of the above dispersion was loaded onto a glassy carbon electrode of 3 mm in diameter. Linear sweep voltammetry with a scan rate of 5 mV s^−1^ was conducted in 0.1 M KOH solution (purged with oxygen for 20 min) using Ag/AgCl (3 M KCl) electrode as the reference electrode and the platinum wire as the counter electrode. The measured potentials vs Ag/AgCl (3 M KCl) were converted to the reversible hydrogen electrode (RHE) scale according to the Nernst equation:

1 $$ {E}_{\mathrm{RHE}}={E}_{\mathrm{Ag}/\mathrm{AgCl}}+0.059 PH+{E^0}_{\mathrm{Ag}/\mathrm{AgCl}} $$

where *E*_Ag/AgCl_ is the experimentally measured potential vs Ag/AgCl reference and *E*^0^_Ag/AgCl_ = 0.21 V at 20 °C [18]. The values of potential provided along the text are referenced against RHE unless otherwise stated.

The apparent number of electrons transferred during ORR was determined by the Koutechy−Levich equation given by:

2 $$ \frac{1}{J}=\frac{1}{J_L}+\frac{1}{J_K}=\frac{1}{{B\upomega}^{1/2}}+\frac{1}{J_K} $$

3 $$ \mathrm{B}=0.62\mathrm{nF}{C}_0{\left({D}_0\right)}^{2/3}{v}^{1/6} $$

where *J* is the measured current density, *J*_*K*_ is the kinetic current density, *J*_*L*_ is the diffusion-limited current density, *ω* is the electrode rotation rate, *F* is the Faraday constant (96,485 C mol^−1^), *C*_0_ is the bulk concentration of O_2_ (1.2 × 10^−3^ mol L^−1^), *D*_0_ is the diffusion coefficient of O_2_ (1.9 × 10^−5^ cm^2^ s^−1^), and *ν* is the kinetic viscosity of the electrolyte (0.01 cm^2^ s^−1^) [18].

## Result and Discussion

**Fig. 1:**
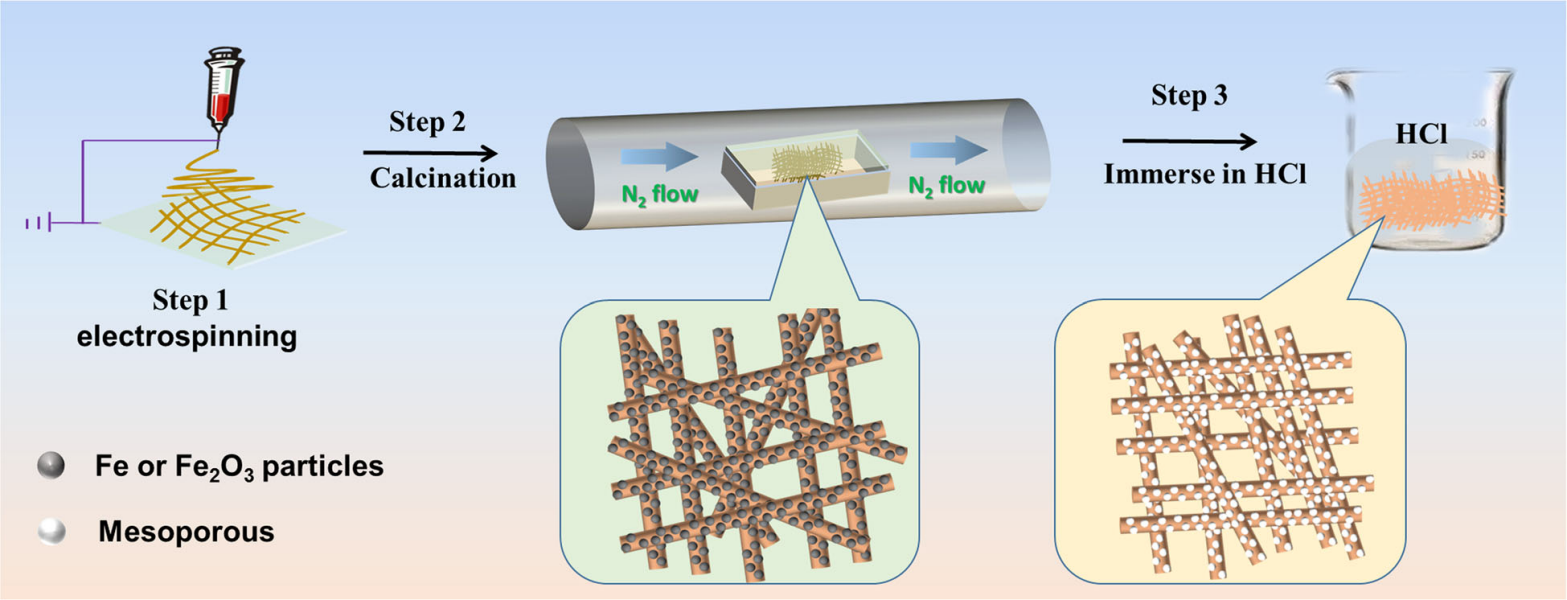
Illustration of the Fe-N-C mesoporous nanofiber preparation steps

The Fe-N-C mesoporous nanofibers were prepared by electrospinning, carbonization, and subsequently HCl immerse process. Figure 1 illustrates the overall preparation scheme for the catalyst. Firstly, precursor solution containing polymer, FeCl_3_ (Fe source), and urea (N source) was prepared and then followed by the electrospinning process, and the precursor nanofibers were obtained; it was transferred into the tube furnace to carbonize the polymer; it should be noted that to pretend the urea volatile under high temperature, a coverage was covered on the top of the crucible; soon afterwards, the obtained black powder was immerse in HCl solution for 5 days to remove the excess metal particle, and then, the Fe-N-C mesoporous nanofibers were obtained (named FN-800).

**Fig. 2:**
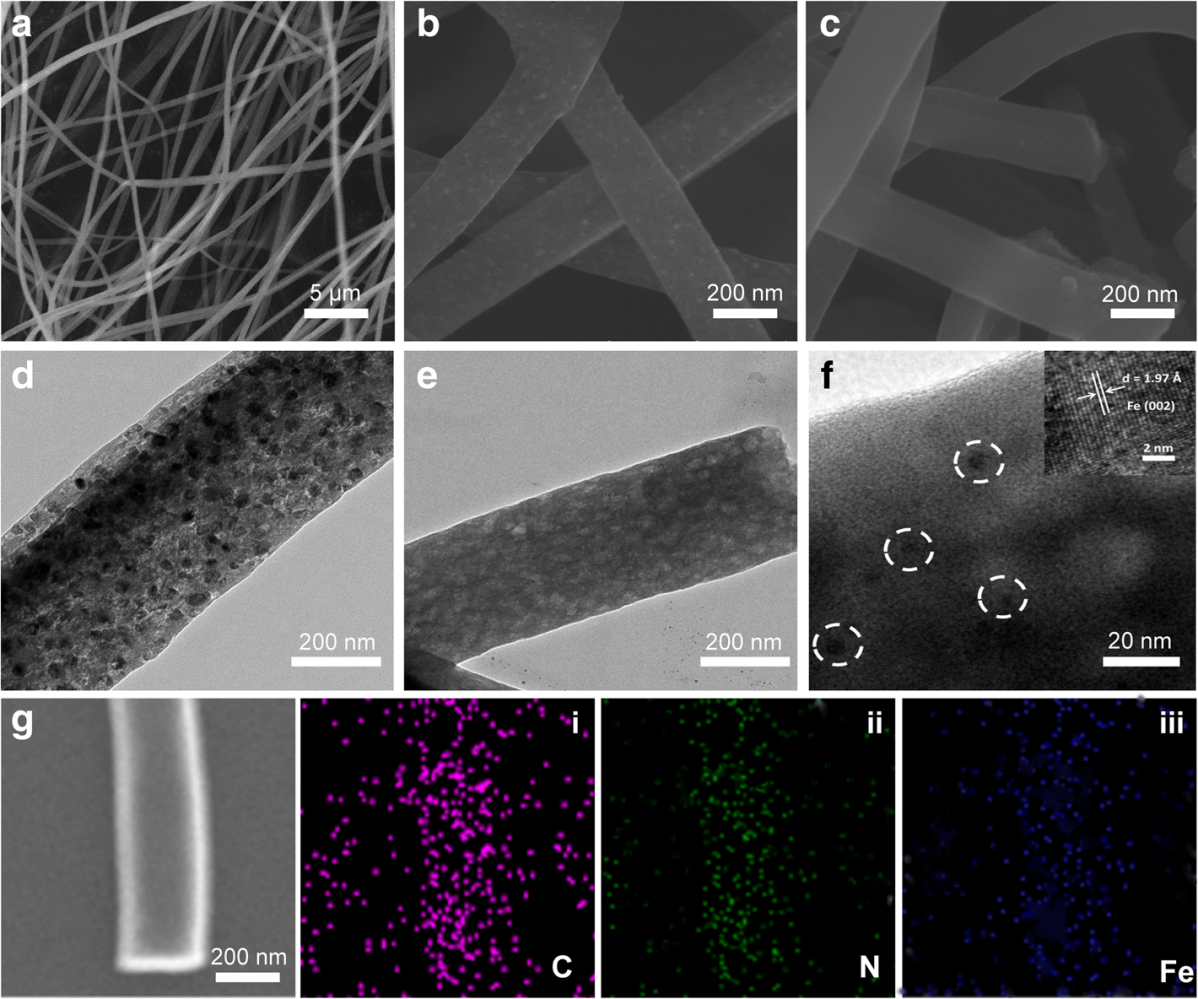
SEM images of FN-800: before calcination (**a**), calcination in 800 °C (**b**), and with acid immerse (**c**). TEM images of FN-800: calcination in 800 °C (**d**); with acid immerse (**e**, **f**). The inset of **f** is HERTEM of the white circle area. SEM and corresponding element mapping image of FN-800 (**g** SEM; **i** C; **ii** N; **iii** Fe)

Figure 2a–c corresponds to the nanofiber morphology evolution during the three stages of preparation process, respectively. As shown, the precursor nanofiber from electrospinning was longer than several tens of micrometers and the diameter is about 500 nm (Fig. 2a). After calcination, the diameter decreased to about 200 nm; meanwhile, a lot of particles were found inlaid in the nanofibers (Fig. 2b), and the TEM further suggest the abundant content both in the surface and inner (Fig. 2d). They are formed by high concentration of iron in the precursor, which have great surface energy at high temperatures and easy to agglomeration. Figure 2c is the SEM image of the sample with acid treat. Clearly, the iron particles on the surface of the nanofibers were disappeared, and the TEM suggest metal particles inside the nanofibers can be removed too (Fig. 2e); moreover, it also reveals the final porous structure of Fe-N-C material. Besides, several particles with a diameter of about 5 nm were found in the nanofibers under high magnifications, an atomic spacing (0.197 nm) was distinguished by HRTEM (insert of Fig. 2f), which could be ascribed to the (002) lattice fringes of tetragonal phase Fe (JCPDS 34-0529). The residual iron is beneficial to catalysis, and it also suggests the good stability. EDX spectra reveal the sample was constructing by Fe, N, C, and O. The atomic ratio was 0.78, 0.53, 95.21, and 3.48%, respectively (Additional file 1: Figure S1). It suggests that although a large amount of metal has been removed, a lot still leaves. The EDX mapping image indicates the Fe and N elements were uniformly distributed in the nanofiber (Fig. 2g, i–iii).

**Fig. 3:**
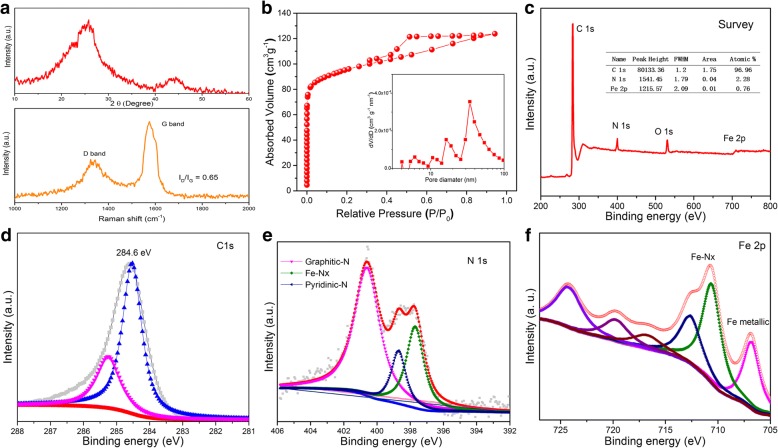
XRD and Raman spectra of FN-800 (**a**, top and **a**, down). N_2_ sorption isotherms and of FN-800 (**b**, the inset corresponds to pore diameter distribution). XPS spectra of FN-800: survey (**c**), C 1s (**d**), N 1s (**e**), and (**f**) Fe 2p

The phase and crystallinity of the FN-800 were determined by XRD as shown in Fig. 3a—top. The peaks at 2*θ* of 26° and 44.5° correspond to the (002) and (100) diffraction peak of graphite (JCPDS 06-0675) [19]; it indicates the graphitic nature. No obvious peaks attributable to Fe could be observed; it should be the result from the low content (0.78%) and uniform dispersion. Furthermore, the Raman spectrum was accompanied to investigate the structure and quality of the carbon materials (shown in Fig. 3a—down). Clearly, the G band was higher than the D band and the *I*_D_/*I*_G_ ratio is 0.65 which indicate the highly graphitized features. The Raman spectrum of N-800 (without FeCl_3_) was also displayed in Additional file 1: Figure S2, which suggest an *I*_D_/*I*_G_ ratio that is 1.06. The result indicates that the introduction of FeCl_3_ could be catalytic to formation of more ordered graphitic carbon, which is helpful for stability and charge transfer. Similar phenomenon was found in other work [19].

The surface area and porous nature of FN-800 were assessed by N_2_ absorption and desorption analysis (Fig. 3b). The remarkable hysteresis loops of type IV indicated the mesoporous structure, which displays a BET surface area (354 m^2^ g^−1^) and the average pore diameter 35.9 nm that indicates a mesoporous type (shown in the insert). The data of FN-800 which was without acid treat was also collected and shown in Additional file 1: Figure S3, and a BET surface area 140 m^2^ g^−1^ was recorded; more than 1.5 times growth of surface area was derived from these porous structures. Without doubt, large surface area could expose more active site and contact with reactant during catalytic process which is benefit for the ORR process.

XPS measurements were conducted to elucidate the chemical composition and element bonding configurations in the Fe-N-C mesoporous nanofibers. The survey spectrum of FN-800 revealed the presence of C (96.96 at%), N (2.28 at%), and Fe (0.76 at%) elements (Fig. 3c and insert table). High-resolution XPS spectra of C 1s spectra were shown in Fig. 3d, which presents two peaks located at 284.6 and 285.4 eV, respectively. The C standard position peak was derived from graphitic, and the peak of higher energy position may attribute to bonding C such as Fe-C and C-N. The N 1s spectra (shown in Fig. 3e) could be fitted into three peaks which are assignable to the pyridinic N (398.7 eV), graphitic N (400.6 eV), and Fe-Nx sites (397.7 eV) [20–23], respectively. The graphitic N was reported to play a crucial role in oxygen reduction; besides, pyridinic N and pyrrolic N can serve as metal coordination sites due to their lone-pair electrons. These three kinds of ORR active nitrogen are of high content in our FN-800 elecrocatalyst [22, 23]. The Fe 2p spectrum is shown in Fig. 3f. The peak at 707.2 eV is suggestive of the presence of metallic iron; the peak at 712.9 eV, 717.4 eV, and 724.5 eV should be attributed to oxidized iron species; the peak at 720 eV was a satellite peak; and the peak at 711.2 eV indicates the Fe-N bonding [24, 25], which agrees with the N 1s spectra before.

To investigate how the coverage of the porcelain boat influences on the Fe-Nx formation during carbonization process, another FN-800 sample was also prepared as the same way which just change the carbonization process by removing the cover. The XPS survey scan and N1s high-resolution spectrum of the sample were present in Additional file 1: Figure S4; clearly decrease of the N peak was found in Additional file 1: Figure S4a; and the C, N, and Fe percentage of elements is 97.36, 0.86, and 0.97 respectively; the N element lost about 62% without the coverage. And the N 1s spectra reveal only two peaks assign to the pyridinic N and graphitic N; the Fe-Nx disappeared which corresponds to the higher formation energy. Combined with the nitrogen source (urea), reaction condition, and corresponding characterization data, we proposed that during the reaction process, urea first produces ammonia at lower temperatures (~ 160 °C). If there is no coverage, it will be taken away by the carrier gas (N_2_). The coverage could produce an amines-rich environment in the porcelain boat; ammonia will further form complex compound and then from Fe-Nx sites. Actually, ammonia was also used as nitrogen source to the preparation of Fe-N-C catalyst for ORR [26, 27]. Our result suggests that urea can be used as a cheap nitrogen source to construct Fe-N-C electrocatalyst via simple improvement during the annealing process.

**Fig. 4:**
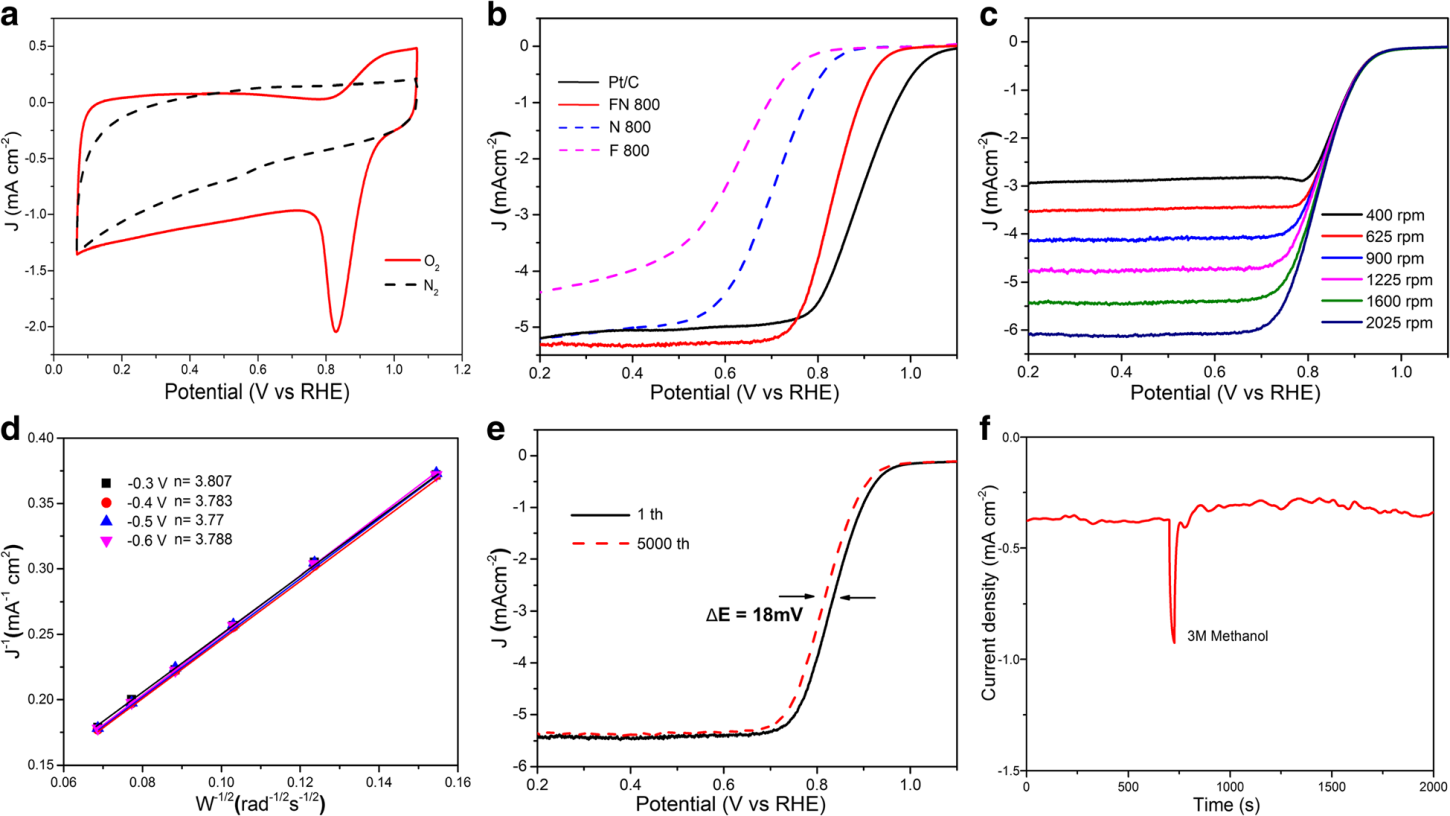
**a** CV curves of FN-800. **b** LSV curves of FN-800, F-800, N-800, and Pt/C in 0.1 M KOH solution. **c** RDE curves of FN-800 at various rotation rates. **d** The corresponding K–L plots (*J*^−1^ vs. *ω*^−1/2^) at different potentials. **e** LSV curves of FN-800 in 0.1 M KOH solution: initial and after 5000 cycles. **f** Methanol tolerance test of FN-800

The electrocatalytic activity of FN-800 was firstly evaluated using cyclic voltammetry, and the result was shown in Fig. 4a; an obvious oxygen reduction peak for samples in the O_2_-saturated solution was observed, whereas no perceptible voltammetry current was found in the presence of N_2_. Linear sweep voltammetry (LSV) curves were obtained with a scan rate of 5 mV/s and a rotating rate of 1600 rpm. As shown in Fig. 4b, the polarization curve of FN-800 displays an onset potential of 0.93 V and a half-wave potential of 0.82 which was close to Pt/C (onset potential of 0.96 V and half-wave potential 0.8 V). The ORR performance is competitive among the reported Fe-N-C and other M-N-C electrocatalyst (Additional file 1: Table S1). As a contrast, F-800 (without N) and N-800 (without Fe) all express poor oxygen reduction ability which indicates the importance of the Fe-Nx species for the ORR in this system. RDE measurements under different rotating speed (Fig. 4c) reveal an electron transfer number of 3.77–3.807 at − 0.30 to − 0.6 V on the basis of Koutecky–Levich (K–L) plots (Fig. 4d), suggesting that the FN-800 catalyst favors a four-electron transfer process toward the ORR and O_2_ is reduced to OH^−^. In contrast, the comparative samples showed a much lower electron transfer number of 1.69–2.07 for F-800 and 1.75–2.43 for N-800, indicating poor electrocatalysis selectivity for these catalysts (Additional file 1: Figure S5). Therefore, the catalysts with different carbonize temperature in the range of 600–1000 °C were also evaluated (Additional file 1: Figure S6) and the highest ORR activity was achieved at 800 °C which was agreed with the previous work [28].

Besides the ORR performance, stability is another key factor for the catalyst. The test result was present in Fig. 4e; FN-800 catalyst exhibits remarkable durability performance, in which the half-wave potential decreases by only ∼ 18 mV after 5000 cycles, with no appreciable variation in the onset potential. It may be because the catalyst is prepared from acid environment. The methanol tolerance test was also made (Fig. 4f). As shown, after the addition of 3.0 M methanol, the ORR current density of FN-800 remains almost the same with negligible change except for a slight oscillation which indicates good methanol tolerance.

## Conclusions

In conclusion, Fe-N-C mesoporous nanofibers with abundant Fe-Nx active sites and large surface area were synthesized via the electrospinning, in situ pyrolysis, and acid treatment process. The use of sealing conditions in the calcination process can effectively improve the nitrogen species content in the catalyst, which is important for improving performance. The as-prepared composite material manifests well electrocatalytic performance for ORR in alkaline electrolyte (onset potential of 0.93 V and half-wave potential of 0.82 V); meanwhile, the electrocatalyst expresses good stability and methanol tolerance. This work may provide new thought for developing high-performance ORR electrocatalysts.

## Additional file

Additional file 1: **Figure S1.** EDX specter of FN-800 and the insert was the element ratio of C, N and Fe, respectively. **Figure S2.** Pore size distributions for FN-800. **Figure S3.** N2 absorption and desorptionof FN-800 without acid treat. **Figure S4.** XPS survey scan and N1 s high resolution spectra of FN-800 which uncover during carbonization process. **Figure S5.** Polarization curves at various speeds and a scan rate of 5 mV/s: (a) N-800; (b) F-800; K-L plots (J^− 1^ vs. ω^-1/2^) at different potentials of N-800 (c) and F-800 (d). Figure S6. LSV of the Fe-N-doped carbon nanofibers catalysts with different carbonize temperature in the range of 600–1000 °C. **Table S1.** Comparison of the ORR performance between FN-800 and other reported catalysts in 0.1 M KOH electrolyte. (PDF 843 kb)

## Abbreviations

DMF: *N*-*N*-Dimethylformamide
EDS: Energy dispersion spectra
MOFs: Metal–organic frameworks
ORR: Oxygen reduction reaction
PAN: Polyacrylonitrile
SEM: Scanning electron microscope
TEM: Transmission electron microscope
XPS: X-ray photoelectron spectra
XRD: X-ray diffraction patterns
