# Supplementary material for: Highly Active and Stable Fe-N-C Oxygen Reduction Electrocatalysts Derived from Electrospinning and In Situ Pyrolysis
Source: Nanoscale Res Lett. 2018 Jul 20;13:218. doi: 10.1186/s11671-018-2635-x (PMC6054600; doi:10.1186/s11671-018-2635-x)
Supplement: Supplementary file 1 — Figure S1. EDX specter of FN-800 and the insert was the element ratio of C, N and Fe, respectively. Figure S2. Pore size distributions for FN-800. Figure S3. N2 absorption and desorptionof FN-800 without acid treat. Figure S4. XPS survey scan and N1 s high resolution spectra of FN-800 which uncover during carbonization process. Figure S5. Polarization curves at various speeds and a scan rate of 5 mV/s: (a) N-800; (b) F-800; K-L plots (J− 1 vs. ω-1/2) at different potentials of N-800 (c) and F-800 (d). Figure S6. LSV of the Fe-N-doped carbon nanofibers catalysts with different carbonize temperature in the range of 600–1000 °C. Table S1. Comparison of the ORR performance between FN-800 and other reported catalysts in 0.1 M KOH electrolyte. (PDF 843 kb) [file 11671_2018_2635_MOESM1_ESM.pdf]

Supplementary Information for

Highly active and stable Fe-N-C oxygen reduction electrocatalysts derived from electrospinning and in-situ pyrolysis

Xuelian Yan[1], Yucen Yao[1], Yuan Chen[2 *].

[1] Research Institute for New Materials Technology, Chongqing University of Arts and Sciences, Yongchuan, Chongqing 402160, PR China

[2] Suzhou institute for energy and material innovations, Soochow University, Suzhou 215006, China

[*] Corresponding author. Tel.: +86-023-49891752.

E-mail: chenyuan216@126.com.

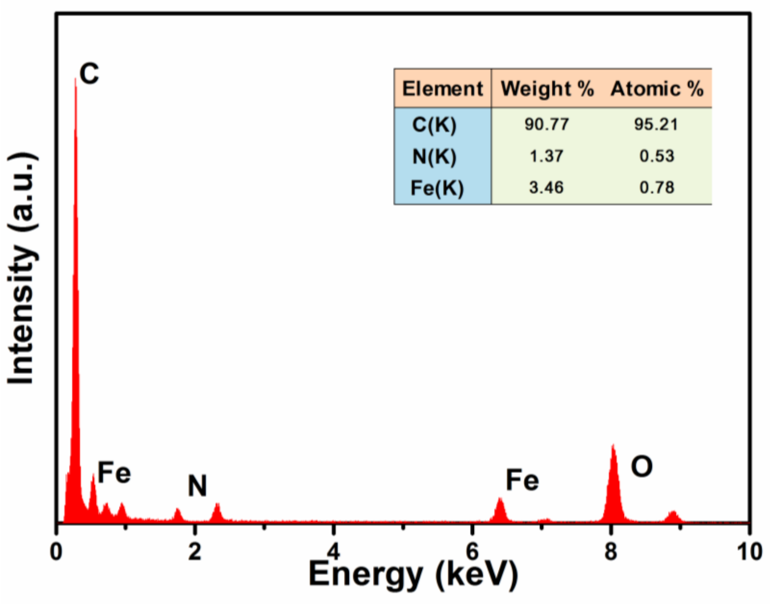

Figure S1. EDX spectre of FN-800 and the insert was the element ratio of C, N and Fe, respectively.

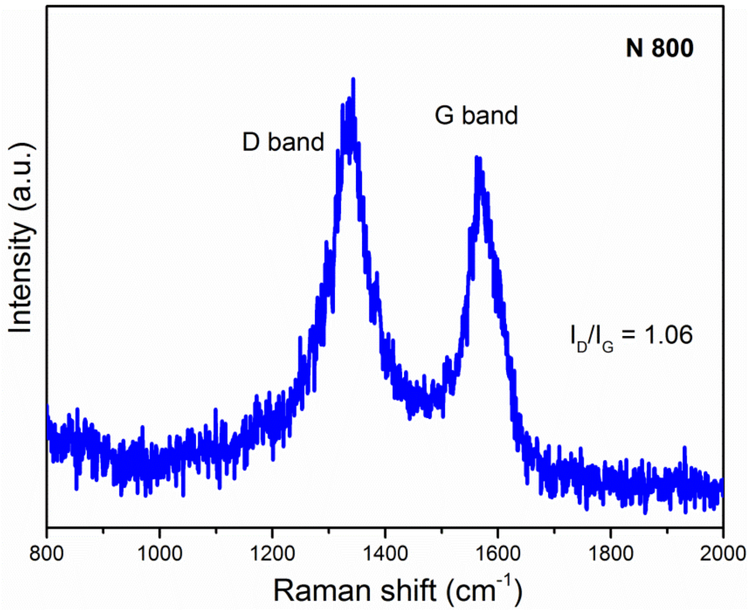

Figure S2. Pore size distributions for FN-800.

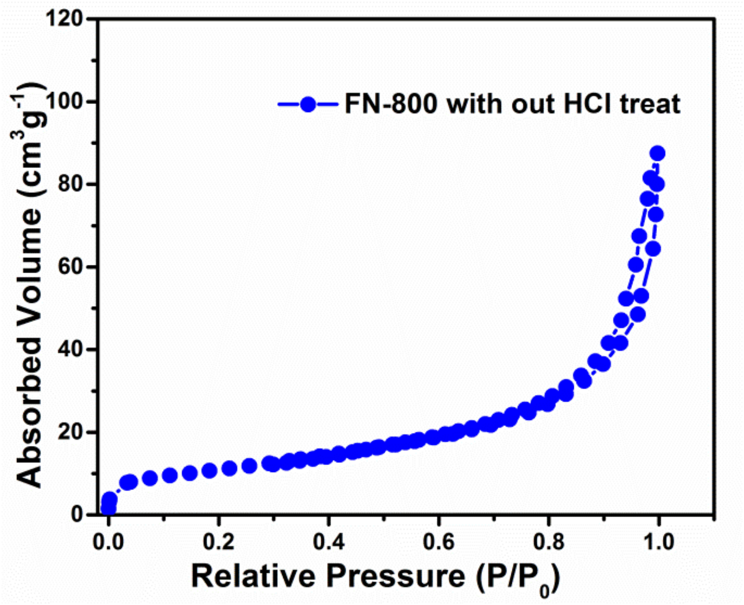

Figure S3. N_2 absorption and desorption of FN-800 without acid treat.

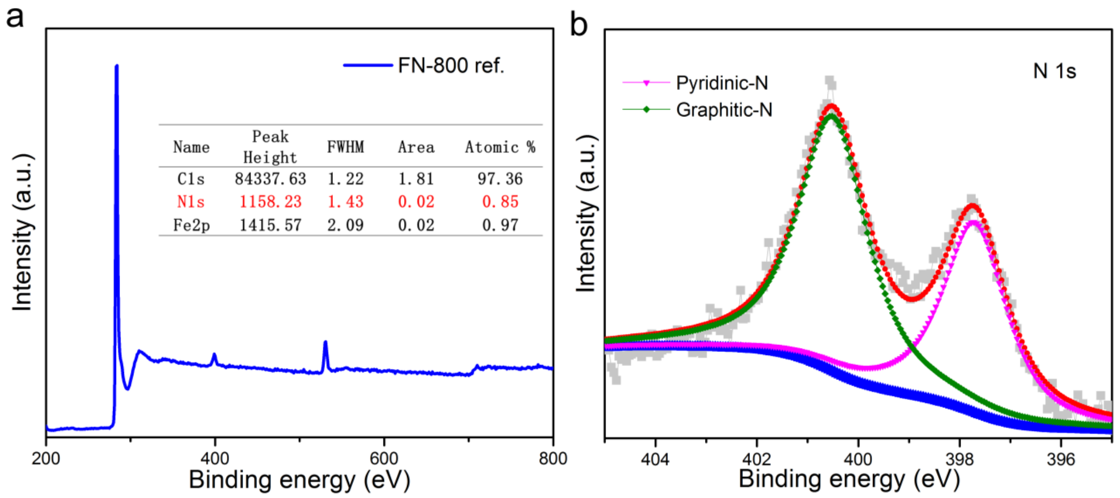

Figure S4. XPS survey scan and N1s high resolution spectra of FN-800 which uncover during carbonization process.

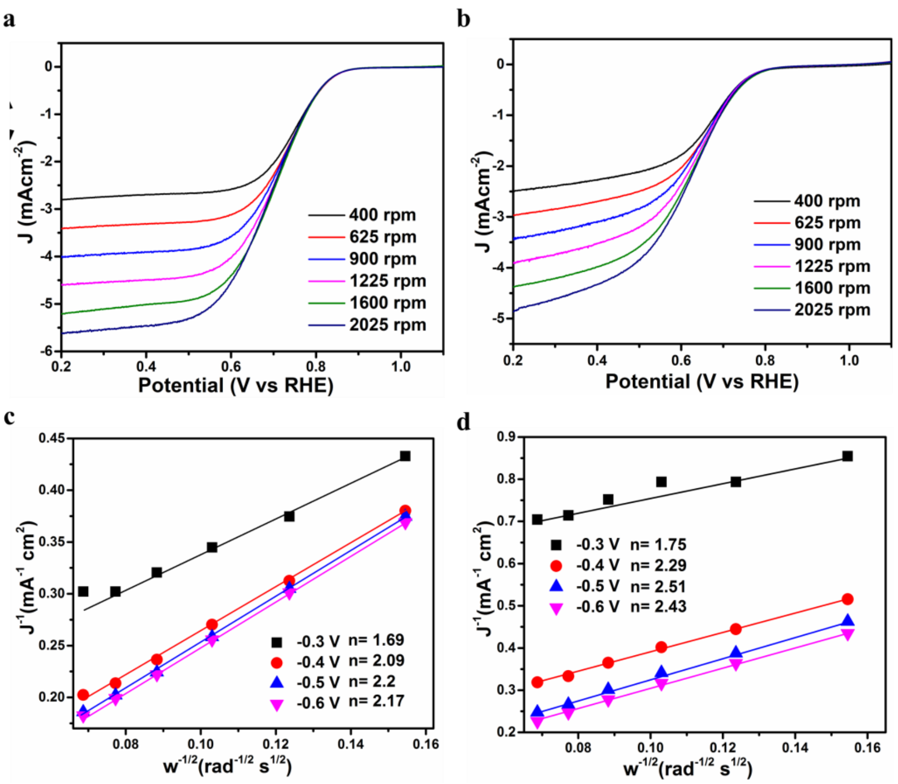

Figure S5. Polarization curves at various speeds and a scan rate of 5 mV/s: (a) N-800; (b) F-800; K-L plots (J^{-1} vs. $\omega^{-1/2}$) at different potentials of N-800 (c) and F-800(d).

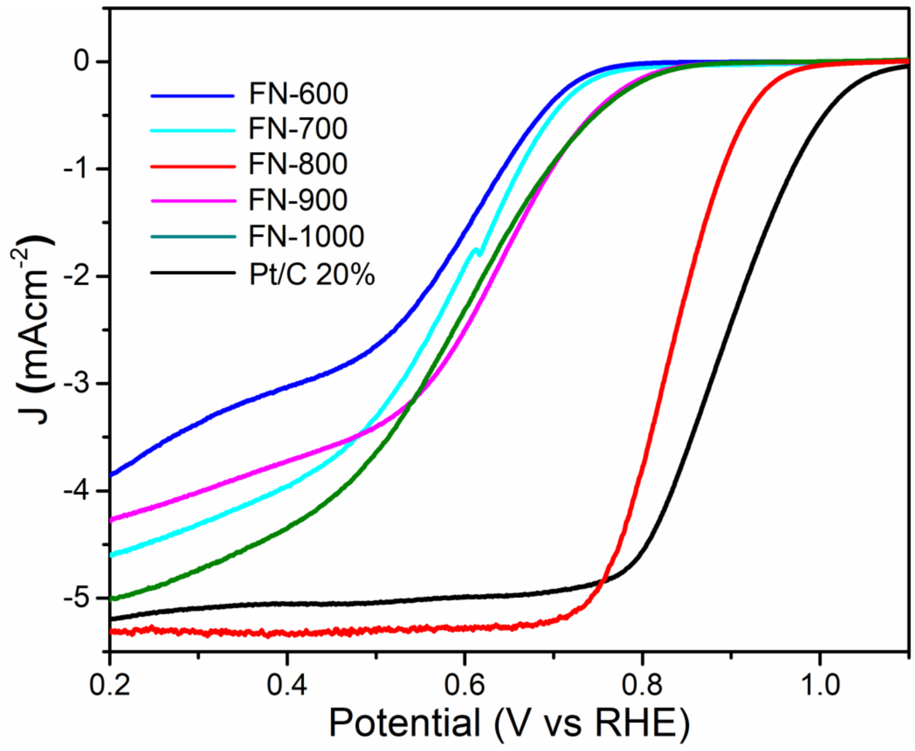

Figure S6. LSV of the Fe-N-doped carbon nanofibers catalysts with different carbonize temperature in the range of 600–1000°C.

Catalyst	Onset potential (V vs RHE)	Half-wave potential (V vs RHE)	Reference
Fe-N-C mesoporous nanofibers	0.93	0.82	This work
Fe-N-doped mesoporous carbon microspheres	1.03	0.86	Adv. Mater. 2016, 28, 7948-7955
Fe–N-doped graphene aerogels	0.97	0.82	Adv. Funct. Mater. 2016, 26, 5708-5717
Fe_3C@ Fe-N-doped graphene	0.98	--	ACS Appl. Mater. Inter. 2015, 7, 21511-21520
Fe-N doped hollow carbon-nanoshells	0.98	0.85	ACS Catal. 2015, 5, 3887-3893
N-doped carbon cubes	0.92	0.8	Nanoscale, 2017, 9, 1059.
CoO@Co/N-rGO	0.95	0.81	J. Mater. Chem. A, 2017,5,5865
NCNT/CoO-NiO-NiCo	0.97	0.83	Angew.Chem. Int.Ed. 2015, 54,9654
CF-NG-Co	0.97	0.85	J. Mater. Chem. A, 2018,6,489
N-CG–CoO	0.9	0.81	Energ Environ Sci. 2014, 7, 609

Table S1. Comparison of the ORR performance between FN-800 and other reported catalysts in 0.1 M KOH electrolyte.
